# The Influence of Phenylalanine Fluctuations and Intake on a 24 h Sapropterin Responsiveness Test in Patients with Phenylketonuria

**DOI:** 10.3390/children12050541

**Published:** 2025-04-24

**Authors:** Ana Jaqueline Nunes, Bianca Fasolo Franceschetto, Lisiane da Gama, Soraia Poloni, Lilia Farret Refosco, Tassia Tonon, Vaneisse Monteiro, Rafael Hencke Tresbach, Fernanda Sperb-Ludwig, François Maillot, Ida Vanessa Doederlein Schwartz

**Affiliations:** 1 Graduate Program in Medical Sciences, Universidade Federal do Rio Grande do Sul (UFRGS), Porto Alegre 91509-900, Brazil; ana.jaquelline@gmail.com (A.J.N.); bffranceschetto@hcpa.edu.br (B.F.F.); ldgama@hcpa.edu.br (L.d.G.); 2 Nutrition and Dietetics Service, Hospital de Clínicas de Porto Alegre, Porto Alegre 90035-903, Brazil; spoloni@hcpa.edu.br (S.P.); lrefosco@hcpa.edu.br (L.F.R.); 3 Medical Genetics Service, Hospital de Clínicas de Porto Alegre, Porto Alegre 91035-903, Brazil; tassiatonon@gmail.com; 4 Graduate Program in Genetics and Molecular Biology, Universidade Federal do Rio Grande do Sul (UFRGS), Porto Alegre 91501-970, Brazil; vaneisse@gmail.com (V.M.); fsperb@gmail.com (F.S.-L.); 5 Service de Médecine Interne, University of Tours, 37032 Tours, France; francois.maillot@univ-tours.fr; 6 Nuclimed—Clinical Research Center, Hospital de Clínicas de Porto Alegre, Porto Alegre 90035-007, Brazil; 7 InRaras—Brazilian National Institute on Rare Diseases, Porto Alegre 90035-903, Brazil

**Keywords:** phenylketonuria, responsiveness, BH4, tetrahydrobiopterin, sapropterin

## Abstract

Patients with phenylketonuria (PKU) who retain residual phenylalanine hydroxylase (PAH) activity may benefit from sapropterin dihydrochloride (sapropterin) administration. Objective: To characterize sapropterin responsiveness in patients with PKU and investigate the impact of natural fluctuations in phenylalanine (PHE) levels and variations in PHE intake on sapropterin responsiveness. Methods: Retrospective chart review study. Patients with PKU who underwent the 24 h responsiveness test, including correction for natural PHE fluctuations, were included. Responders were defined as those who exhibited a >30% reduction in PHE levels within 8 h and/or 24 h after intake of 20 mg/kg sapropterin, correcting for the natural fluctuation in plasma PHE levels on day 1. Patients with a 28–30% reduction in PHE were considered sapropterin-responsive only if they had a concordant genotype. Results: Fifteen patients completed the test; however, three were excluded due to non-compliance. Additionally, one patient with mild PKU exhibited a borderline response, but the genotype agreement could not be assessed, rendering the test inconclusive. The rate of responsiveness could be assessed for eleven patients (six mild and five classical PKU). Among the patients with mild PKU, four were classified as responders: three at both 8 h and 24 h (reduction in plasma PHE: −75.9 ± 20.2% at 8 h and −75.7 ± 37.0% at 24 h) and one at 8 h only (reduction in plasma PHE: −28.7%). All patients with classic PKU (*n* = 5) were non-responders. Conclusions: A 24 h sapropterin responsiveness test incorporating correction for natural fluctuations in PHE levels identified a proportion of sapropterin-responsive patients with mild PKU similar to that described in the literature. PHE consumption should be objectively controlled during the protocol to avoid bias in determining responsiveness.

## 1. Introduction

Phenylketonuria (PKU) is an autosomal recessive disease caused by pathogenic variants in the gene *PAH*, which encodes the hepatic enzyme phenylalanine hydroxylase (PAH). The absence or deficient activity of this enzyme prevents the hydroxylation and conversion of phenylalanine (PHE) into tyrosine (TYR), a reaction dependent on the cofactor tetrahydrobiopterin (BH4). The buildup of PHE in blood and brain leads to neurotoxicity and irreversible damage, which can only be avoided by timely screening, diagnosis, and treatment [1].

Current guidelines recommend lifelong treatment for PKU, consisting of a PHE-restricted diet that excludes animal protein, restrict high-PHE plant-based foods, and includes PHE-free metabolic formulas [2,3]. The discontinuation of treatment carries a risk of cognitive and behavioral impairment [4,5], and untreated patients present mental health conditions, such as depression, and neurological complications [6]. The administration of sapropterin—in the form of sapropterin dihydrochloride (sapropterin)—is an approved therapy for PKU in many countries [5,7]. Sapropterin is believed to function as a pharmacological chaperone, increasing tolerance to PHE, thereby allowing greater dietary flexibility and improving adherence to the restrict diet [8,9,10]. In the Brazilian public health system, sapropterin is reimbursed only for women of childbearing age or in the periconceptional/gestational period who were found to be responsive, due to the risk of PKU embryopathy [5,11]. Patients with mild PKU are those most responsive to sapropterin therapy [12].

Several protocols for identifying sapropterin-responsive PKU individuals have been described; however, the optimal protocol for determining patient responsiveness, as well as the definition of responsiveness itself, remains a matter of debate among experts [8,12]. Natural fluctuations in PHE levels—variations in blood PHE concentrations throughout the day, influenced by factors such as diet, metabolism, and meal timing—as well as variations in PHE intake during test days, are potential confounding factors. Nevertheless, these variables have been poorly explored in existing protocols, and their actual impact on sapropterin responsiveness has not yet been well established. In Brazil, since 2019, the Ministry of Health (MoH) has recommended a 24 h testing protocol, conducted in an outpatient setting without the need for hospitalization, which takes into account the natural fluctuations in PHE levels [5]. The present study aims at evaluating the sapropterin responsiveness of patients with PKU undergoing the Brazilian MoH 24 h short test and assessing the impact of natural PHE fluctuations and variations in PHE intake on sapropterin response.

## 2. Methods

This was a retrospective chart review study approved by the Research Ethics Committee at Hospital de Clínicas de Porto Alegre—HCPA, Brazil (35624720.5.0000.5327).

### 2.1. Population

**Figure 1:**
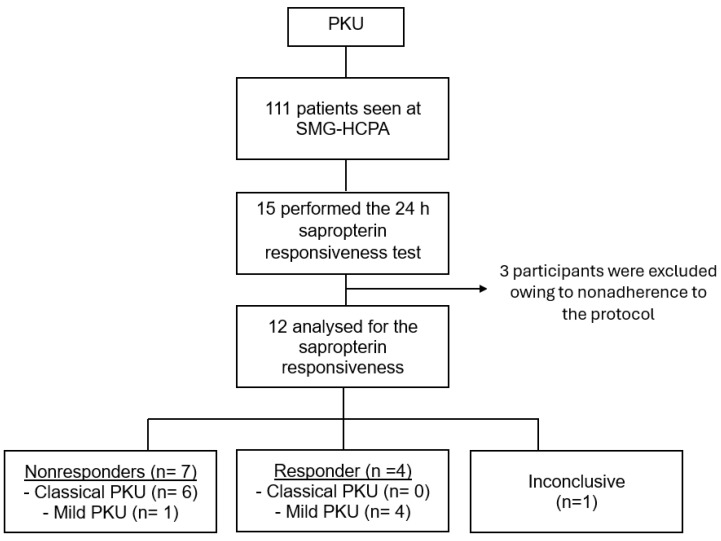
Patient flowchart: inclusion, exclusion, and study results summary. PKU = phenylketonuria; SMG-HCPA = Serviço de Genética Médica do Hospital de Clínicas de Porto Alegre, Brazil.

PKU patients seen at the Medical Genetics Service—HCPA (*n* = 111) had their files reviewed, and patients who had performed the 24 h sapropterin responsiveness test recommended by the Brazilian MoH were included in the study (Figure 1).

Fifteen unrelated patients were included in the study. The responsivity was not evaluated if the variation in daily PHE intake during the test was >30%.

### 2.2. The 24 h Sapropterin Responsiveness Test

This test is a three-day outpatient test, covering a 24 h period after sapropterin intake [5]. On day 1 (D1), the natural fluctuation of plasma PHE is evaluated by measuring PHE levels at baseline or 8 am (Point 0, D1) and at 4 pm (Point 1, D1). On day 2 (D2), PHE levels are measured at 8 am (this point is both Point 2, D1, and Point 0, D2), followed immediately by the intake of a single 20 mg/kg dose of sapropterin, diluted in water or industrialized apple juice. PHE levels are then measured at 4 pm (Point 1, D2) and at 8 am at D3 (this point is both Point 2, D2, and Point 0, D3). Patients fast for 8 h before morning blood drawing and for 1 h before the afternoon blood drawing. High-performance liquid chromatography (HPLC) is used to measure plasma PHE.

### 2.3. Sapropterin Responsiveness

Two criteria were established to define responsiveness, both of which consider the percent natural fluctuation in PHE levels, as assessed on D1:

Criterion 1—a ≥30% reduction in PHE within 8 h of sapropterin intake, considering the percent natural fluctuation of D1. For this point, the natural fluctuation of D1 was considered to be the difference in PHE levels between P1, D1, and P0, D1; and/or

Criterion 1—a ≥30% reduction in PHE at 24 h after sapropterin intake, considering the percent natural fluctuation of D1. For this point, the natural fluctuation of D1 was considered to be the difference in PHE levels between P2, D1, and P0, D1.

The formula used to evaluate responsiveness considers the percent variation in PHE levels at 8 h and 24 h after sapropterin intake, i.e., from baseline at D2 (P0D2 or P2D1), subtracting the percent fluctuation measured on D1 [5]:

8 h: [((P1D2 − P0D2/P0D2 × 100) − ((P1D1 − P0D1/P0D1 × 100)]

24 h: [((P2D2 − P0D2/P0D2 × 100) − ((P2D1 − P0D1/P0D1 × 100)]

where P0D1 = first blood draw on D1; P1D1 = second blood draw on D1; P2D1 or P0D2 = first blood draw on D2, prior to sapropterin administration; P1D2 = second blood draw on D2, 8 h after sapropterin administration; and P2D2 = single blood draw on D3, 24 h after sapropterin administration.

Patients with a reduction of between at least 28–30% at 8 h and/or 24 h (i.e., borderline response) were considered responders in the presence of a concordant genotype. The test was considered inconclusive or invalid if the patient was non-adherent to the test protocol (failure to take all medication and/or variation in PHE intake > 30% between test days). If the patient exhibited a satisfactory response to sapropterin even with PHE intake ≥ 30% in relation to the previous test day, the genotype responsiveness criterion was used (as described below in the section “Agreement of Responsiveness”).

### 2.4. Dietary Intake

Patients who performed the test were instructed to adhere to their usual PHE-restricted diet and take their metabolic formula as prescribed to avoid any fluctuations in PHE intake. Adherence to the diet was assessed by food records in standardized forms completed by the patient during the 3 test days. To enhance the accuracy and reliability of the data, the records were complemented by the analysis of photographs of the meals consumed on the three test days. On the day before the start of the test (day 0 or D0), dietary records started using a standardized form. PHE, energy, and protein intakes were calculated using Nutribase™ software, v.16.21(NB16Cloud, Cybersoft Inc., Phoenix, AZ, USA), which defaults to the USDA food table.

### 2.5. Genotypes

Genotype data were obtained through a review of medical records or as previously published in the work of Tresbach et al. (2020) [13].

### 2.6. Phenotype Determination

The type of PKU was classified according to the plasma PHE level at diagnosis and/or according to the patient’s PHE tolerance over the years on dietary management alone, as described by Nalin et al. (2011) [14]. Cutoff points at diagnosis were as follows [5]: PHE > 1200 µmol/L = classic PKU; PHE 360–1200 µmol/L = mild PKU; and PHE 120–360 µmol/L = non-PKU hyperphenylalaninemia.

### 2.7. Agreement of Responsiveness

The agreement of responsiveness to sapropterin with the published literature was assessed by comparing each patient’s genotype with the information contained in the BioPKU database (http://www.biopku.org) (accessed on 12 June 2024) [15] for that genotype. Patients whose genotype was recorded in more than five tested individuals in the database, the majority (>50%) of whom had the same response to sapropterin, were considered to have concordant responsiveness.

### 2.8. Statistical Analyses

Data were compiled in a Microsoft Excel^®^ 2013 spreadsheet and analyzed in the SPSS Version 21.0 software environment (IBM Corp, Armonk, NY, USA). Normally distributed continuous variables were described as means and standard deviations, while asymmetrically distributed variables were expressed as medians and interquartile ranges. Categorical variables were described as absolute (*n*) and relative (%) frequencies and compared using Fisher’s exact test. Normally distributed continuous variables were compared using Student’s *t*-test, while asymmetrically distributed ones were compared through the Mann–Whitney U test. Spearman coefficients were used to test for correlation between nonparametric continuous variables. The assumption of normality was verified using the Kolmogorov–Smirnov test, and the significance level (1–α) was 95%.

## 3. Results

Out of the 15 patients included (10 females; median age of 8 years, interquartile range 7–10 years), 7 had classic PKU (46.7%) and 8 had mild PKU (53.3%). Three children were excluded owing to non-adherence to the established protocol: one patient with classic PKU was unable to take all the medication and two (one each with mild PKU and classic PKU) had a >30% variation in PHE intake between test days. The flow chart of the study is shown in Figure 1.

### 3.1. Sapropterin Responsiveness

Of the twelve patients whose tests were validated, one patient with mild PKU (pt. 12, Table 1) showed a borderline response, with a −29.1% reduction in plasma PHE at 8 h; however, the test was deemed inconclusive due to the impossibility of evaluating genotype–phenotype agreement. As a result, the sapropterin responsiveness was established for 11 patients (Figure 1; classic PKU = 5, mild = 6): 66.7% (4/6) of those with mild PKU were classified as responders and all classic PKU patients were non-responders. Three patients (pt. 1, 2 and 5, Table 1) were responsive at 8 h and 24 h (reduction in plasma PHE: −75.9 ± 20.2% at 8 h and −75.7 ± 37.0% at 24 h). One patient (pt. 3, Table 1) was responsive only at 8 h, with a −28.7% reduction, and was considered borderline responsive after genotype analysis. Plasma PHE levels and the percent reduction at each time point after sapropterin administration for each patient included in the analyses are described in Table 1.

Responder and non-responder patients did not differ regarding age, sex, and type of PKU (*p* = 0.471, *p* = 0.559, and *p* = 0.103, respectively).

### 3.2. Phenylalanine Intake

**Figure 2:**
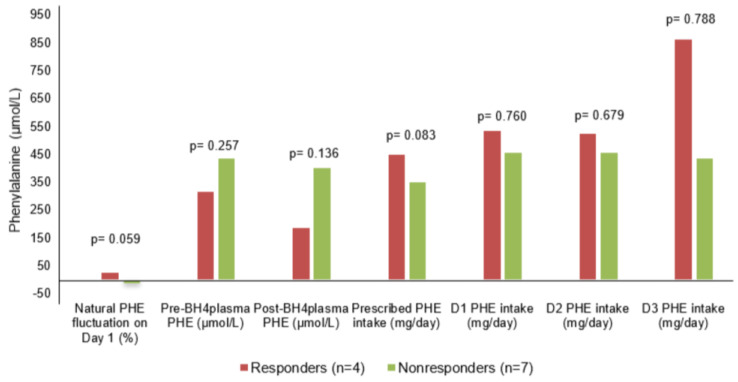
Natural PHE fluctuation on day 1 (%), plasma PHE before and after sapropterin intake (μmol/L), and PHE intake (mg/day) during the sapropterin test (*n* = 11). One patient had an inconclusive test result and was not included in either the responder or non-responder groups. PHE = phenylalanine; BH4 = sapropterin dihydrochloride; D1 = day 1; D2 = day 2; D3 = day 3.

Regarding PHE intake on test days, the median variation in plasma PHE between D0 and D1 was 0 μmol/L (IQR −25.00; 42.8) and again 0 μmol/L (−17.39; 12.96) between D1 and D2. This variation in intake between days 0 and 1 did not show any correlation with PHE levels at time points 1, 2, or 3 before sapropterin administration (*p* = 0.760; *p* = 0.679; *p* = 0.788, respectively), and the variation in intake between days 1 and 2 also did not correlate with plasma PHE at time points 4 and 5 (*p* = 0.413 and *p* = 0.733, respectively). Data on natural fluctuation in PHE, PHE intake on test days, and plasma PHE before and after sapropterin administration for the patients included in the analyses are shown in Figure 2 (*n* = 11).

### 3.3. Agreement of Responsiveness

As described in Table 1, all participants had been genotyped, and all nine patients (100%) in whom agreement could be assessed had responses concordant with those described in BioPKU [15]. Patient 8’ data have not yet been entered into BioPKU, while the genotypes of patients 7 (c.[754C>T];[1222C>T] and 12 (c.[1042C>G];[1066-11G>A]) were present in only four other patients tested in the database, precluding a classification of agreement. If the natural fluctuation of PHE levels was not considered, agreement would be 66.7% (six out of nine patients).

### 3.4. Natural Fluctuation of Phenylalanine Levels

The median natural fluctuation of PHE levels was 5.9% (IQR −15.8; 16.8) overall, 26.1% (IQR −0.8; 34.1) in responders, and −2.6% (IQR −22.5; 10.4) in non-responders, with no statistically significant difference between the two groups (*p* = 0.059), as shown in Figure 2. Each patient had their results analyzed both considering the natural fluctuation of PHE (according to the test protocol) and without considering this (i.e., analyzing only the variation in PHE 8 h and 24 h after sapropterin administration). If the natural variation in PHE had not been considered in the test protocol, it would have failed to identify one patient with mildly sapropterin-responsive PKU, as shown in Table 1 (pt. 5).

### 3.5. Adverse Events

One adult patient developed dyspepsia after the administration of sapropterin. The test was discontinued, but her responsiveness was assessed at 8 h, and she was a non-responder, as shown in Table 1 (pt. 11).

## 4. Discussion

The objective of this study was to describe the use of a short (24 h) protocol to identify sapropterin responsiveness in patients with PKU and to evaluate the interference of the natural fluctuation in PHE, and of PHE intake on test days. The response found in this study was similar to that reported in the literature [8,16,17,18], and the agreement between actual responsiveness and genotype-predicted responsiveness was 100%, exceeding the 71% rate reported by Wettstein et al. (2015) in a study with 4181 patients with PKU [18].

With this protocol, we were able to identify patients with mild PKU who were responsive according to both criteria and only one who was borderline responsive at 8 h. This is consistent with the literature [8,14,16,17,19], in that patients with mild PKU are known to respond better to sapropterin as compared to classic PKU. A study published in 2011 by Anjema, K., et al. [19] demonstrated that 16% of responsive patients could be missed by not evaluating response to sapropterin at 48 h; however, in this protocol, sapropterin was administered twice, with a 24 h interval. In an article published by a panel of experts [20], the authors recommend that patients with plasma PHE levels within the target range be tested with a 48 h protocol, which can be prolonged for up to 4 weeks in patients who do not respond; the objective of these tests lasting ≥48 h would be to identify late responders. Since validated, published protocols found a response rate of approximately 30 to 50%, especially in patients with mild PKU; the 24 h screening protocol utilized herein found a similar response rate and identified patients who respond faster (within 24 h), without the burden of a prolonged test. Patients who demonstrated a trend toward reduction in PHE levels between 8 h and 24 h could have their responsiveness confirmed in a prolonged test (4 weeks to 6 months) [19,20]

Unlike other responsiveness protocols, the short test described in our study considers the natural fluctuation in PHE levels, while others only use the difference between pre- and post-sapropterin plasma PHE. Taking natural PHE fluctuations into account appears to increase the accuracy of the test and make it more cost-effective than longer test protocols, especially when implemented at a nationwide level by health systems. In this sample of patients, if we had not considered natural fluctuations, the response rate would have been five out of twelve patients.

PHE intake is a variable that is often poorly controlled in studies, and we believe that significant variations in intake may affect test results. We adopted an empirical cutoff limit of 30% and this should be taken into consideration especially if there is a ≥30% reduction in intake, particularly between D1 and D2, when sapropterin is administered. The 30% limit adopted in our protocol proved to be appropriate, as statistical analysis indicated that variations in PHE intake did not significantly affect the test results.

One unique characteristic of our study was that we controlled for PHE intake through both food records and photo recording, which helped prevent any impact on plasma PHE levels. Other protocols [16,17,19] do not mention whether or how control for this variable was carried out. It is worth stressing that, because patients come to the study center from other cities, for some, their habitual eating environment was replaced by restaurants, which led to a greater food intake in general; this was the case with one of the excluded patients, who had the highest dietary intake on the first day of the test and reduced it (in relation to D0) on the day of sapropterin administration, with a >90% difference between days 0 and 1 and a −35% difference between days 2 and 3. We conclude that even when patients are advised not to change their usual diet and stick to their usual prescribed or approved foods, the amount consumed and the consumption pattern can change depending on the environment, which can lead to variations in PHE levels that go unnoticed by caregivers. We thus suggest that the caregivers of PKU patients undergoing sapropterin responsiveness testing be given a standard controlled menu and instructed to follow it on all three test days to avoid the invalidation of results which would require the future repetition of the protocol. Furthermore, photographing meals may help maintain accurate food records, which should then be evaluated together by the dietitian and caregiver to confirm serving sizes, preparation methods, amounts consumed, and detect any snacks/treats that may go unnoticed by the family.

Our study has both strengths and limitations. Regarding the strengths of the study, we assessed the responsiveness to sapropterin considering the influence of natural fluctuations in PHE levels and variations in PHE intake on responsiveness to sapropterin. Furthermore, we also utilized food records in standardized forms verified by analyzing photographs of meals throughout the three test days, used patients’ genotypic data, and described data obtained from the real-life follow-up of PKU patients. Regarding limitations, compared to other studies cited herein, our small sample size can be considered a limitation, particularly due to losses resulting from exclusions. Furthermore, the short test protocol does not allow the identification of late responders (>48 h after sapropterin intake), most of whom are patients with classic PKU. Another limitation was of a geographic nature. Although all patients were from other cities, none showed any unwillingness to travel for the test; however, the change in environment appears to have affected their dietary habits, which ultimately led to the exclusion of two patients. Age would not be a reason to exclude potential candidates as children, as they can cooperate with open dialog about the tests and strategies such as diluting the medicine in industrialized apple juice can be implemented.

## 5. Conclusions

A short (24 h) protocol including correction for natural fluctuations in PHE levels was able to identify a proportion of sapropterin-responsive patients with mild PKU, similar that described in the literature. Correcting for the natural fluctuations in PHE levels seems to identify more responders, but additional studies with this focus and with larger sample sizes are needed to confirm this. PHE consumption should be objectively controlled during the protocol to avoid bias in determining responsiveness.

## Figures and Tables

**Table 1:** Plasma phenylalanine levels and percent reduction thereof after administration of 20 mg/kg of sapropterin dihydrochloride, with and without correction for the natural fluctuation in phenylalanine levels in patients with phenylketonuria.

		Genotype			Responsiveness According to Genotype	Pre-BH4 PHE (µmol/L)			Post-BH4 PHE (µmol/L)		% Change in PHE After BH4, Corrected for Natural Fluctuation *		Responsiveness with Correction for Natural Fluctuation *		% Change in PHE After BH4, not Corrected for Natural Fluctuation **		Responsiveness Without Correction for Natural Fluctuation **	
PT	Type of PKU	Sex	Age (Years)	Allele 1/ Allele 2		P0 D1	P1 D1 (8 h)	P2 D1 (24 h)	P1 D2 (8 h)	P2 D2 (24 h)	8 h	24 h	8 h	24 h	8 h	24 h	8 h	24 h
1	Mild	F	8	c. 106611G>A/ c. 1169A>G	100% (8/8)	297.3	354.0	488.1	231.0	236.5	−71.7	−115.7	R	R	−52.7	−51.5	R	R
2	Mild	F	7	c.1169A>G/ c.12222C>T	100% (25/25)	213.5	287.0	259.0	94.8	135.8	−97.8	−68.8	R	R	−63.4	−47.5	R	R
3	Mild	M	16	c. 194T>C/ c. 1241A>G	82% (9/11)	254.6	235.7	197.6	126.3	155.3	−28.7	−0.9	R	NR	−36.1	−21.4	R	NR
4	Classic	F	10	c. 1042C>G/ c. 1315+1G>A	17% (1/6)	404.0	445.9	456.6	440.7	381.2	−13.8	−29.5	NR	NR	−3.5	−16.5	NR	NR
5	Mild	M	7	c. 194T>C/ c. 1222C>T	55% (11/20)	588.5	783.5	704.1	528.4	541.9	−58.1	−42.6	R	R	−24.9	−23.0	NR	NR
6	Classic	F	13	c. 1222C>T/ c. 1222C>T	3% (3/98)	591.1	649.2	681.8	666.7	621.9	−12.0	−24.1	NR	NR	−2.2	−8.8	NR	NR
7	Classic	F	9	c. 754C>T/ c. 1222C>T	0% (0/4)	303.1	335.2	340.0	329.7	427.7	−13.6	13.6	NR	NR	−3.0	25.8	NR	NR
8	Classic	F	8	c. 712A>C/ c. 814G>T	NA (0/0)	457.4	372.1	322.5	344.2	432.0	25.4	63.4	NR	NR	6.7	33.9	NR	NR
9	Mild	F	4	c. 782G>A/ c. 1315+1G>A	7% (2/30)	523.9	510.0	568.1	509.4	523.3	−7.7	−16.3	NR	NR	−10.3	−7.9	NR	NR
10 ***	Mild	M	7	c. 473G> A/ c. 1162G> A	20% (1/5)	444.1	344.2	418.7	225.1	287.4	−23.7	−25.6	NR	NR	−46.2	−31.3	R	R
11 ***	Classic	F	24	c.782G> A/ c.1315+1G>A	4% (1/25)	844.0	465.9	586.2	393.3	NA	11.9	NA	NR	NA	−32.9	NA	R	NA
12 ****	Mild	F	9	c. 1042C> G/ c.1066-11G>A	0% (0/4)	340.6	347.3	326.7	237.8	315.8	−29.1	0.7	I	NR	−27.2	−3.3	NR	NR

BH4 = sapropterin dihydrochloride; PHE = phenylalanine; PT = patient; NA = not available; F = female; M = male; R = responder; NR = non-responder; P0D1 = first blood draw on day 1; P1D1 = second blood draw on day 1; P2D1 or P0D2 = first blood draw on day 2, before sapropterin; P1D2 = second blood draw on day 2, 8 h after sapropterin; P2D2 = single blood draw on day 3 (D3), 24 h after sapropterin; I = inconclusive. * According to formula correcting for the natural fluctuation in PHE on day 1 (clinical practice guideline); ** No correction for natural fluctuation in PHE on day 1; *** Patients whose responsiveness is discordant with BioPKU if not considering the natural fluctuation in PHE on day 1; **** Patient with borderline response, considered inconclusive due to the small number of tested individuals in the BioPKU database (*n* < 5). Patients 7, 8, and 12 had genotypes with less than 5 entries in the BioPKU database; Patient 11 developed dyspepsia and discontinued the test; Patients 1, 2, 4, 7, 8, 9, and 11 had their genotypes described in previous work by Tresbach et al. [13].

## Data Availability

The data for this work are available in the research group’s database and can be requested by contacting the corresponding author.
